# The Effect of Dye Density on the Efficiency of Photosensitization of TiO_2_ Films: Light-Harvesting by Phenothiazine-Labelled Dendritic Ruthenium Complexes

**DOI:** 10.3390/molecules14103851

**Published:** 2009-09-28

**Authors:** Marye Anne Fox, James K. Whitesell, Douglas Magde, Lin-Yong Zhu

**Affiliations:** 1 Department of Chemistry and Biochemistry, University of California at San Diego, 9500 Gilman Drive, La Jolla, CA 92093-0358, USA; E-Mails: jkw@ucsd.edu (J.K.W.); dmagde@ucsd.edu (D.M.); 2 Institute of Photographic Chemistry, Chinese Academy of Sciences, Beijing 100101, China; E-Mail: linyongzhu@hotmail.com (L.-Y.Z.)

**Keywords:** photosensitization, light-harvesting, ruthenium, dendrimers

## Abstract

A family of dendritic tris-bipyridyl ruthenium coordination complexes incorporating two or four carboxylate groups for binding to a TiO_2_ surface site and another dendritic linker between the metal complex and highly absorptive dyes were formulated as thin films on TiO_2_ coated glass. The family included phenothiazine-substituted dendrons of increasing structural complexity and higher optical density. The dye-loaded films were characterized by steady-state emission and absorption measurements and by kinetic studies of luminescence and transient absorption. Upon photoexcitation of the bound dyes, rapid electron injection into the metal oxide film was the dominant observed process, producing oxidized dye that persisted for hundreds of milliseconds. Complex decay profiles for emission, transient absorption, and optical bleaching of the dendritic dyes point to highly heterogeneous behavior for the films, with observed persistence lifetimes related directly to structurally enhance electronic coupling between the metal oxide support and the dendritic dyes.

## 1. Introduction

Integrated arrays containing photosensitizers, electron donors and acceptors, and semiconductor nanoparticles have been recognized for over a decade as key components of next-generation solar cells [1,2,3,4,5,6,7]. Recently, for example, one such array has been prepared as a key component for biotechnology that photonic applications made possible though integrated arrays [8]. By extending these studies to include self-assembled arrays of increasing complexity, e.g., to dye multi-layers, ever more subtle control of electron migration can be attained. Integrated arrays also offer access to highly aggregated materials with superior optical responses that exceed those attained by the simple monolayer dispersions typically used in photoelectrochemical cells and sensors. It is our intent, here, to study the key absorptive elements of integrated arrays that may be useful in solar energy conversion in order to optimize interfacial charge injection efficiency in a future working cell.

One of the “Holy Grails” of solar energy conversion is the efficient production of hydrogen as a combustible fuel via the solar-induced splitting of water to its component elements [9]. The striking observations of Fujishima and Honda in a series of elegant papers published in the late 1970s showed that ultraviolet or sunlight irradiation of an n-TiO_2_ photoanode connected to a platinum counter-electrode evolved hydrogen and oxygen, albeit with low quantum efficiency, under an applied bias [10,11]. This discovery prompted a broad array of detailed investigations soon thereafter [12].

We now know well the physical processes that ensue upon irradiation of various metal oxide-solution interfaces, but an inherent mismatch of absorption onset of the doped metal oxide surface with the solar spectrum persists, limiting attainable efficiency. Many thoughtful approaches are available that describe the characteristics of an ideal water-splitting cell, and thorough justifications for the extensive use of metal oxides, binary oxides, and twinned oxide configurations have been presented [13]. Even so, observed efficiency remains low, and scale-up remains difficult.

### 1.1. Dye Sensitization

As an alternative to band gap irradiation of metal oxides, Grätzel and coworkers have shown that solar responsiveness can be significantly improved when oxide surfaces are modified by adsorption or covalent attachment of dyes [5,6,7]. Photoexcitation of the dye facilitates electron injection into the conduction band of the metal oxide, thus initiating electron-hole pair formation. The kinetic rate constant for charge injection is at least two orders of magnitude greater photoreduction of phenothiazine. Their observations have prompted an extensive search for an ideal photosensitizer that can be excited by visible light in a range that better matches the solar spectrum, and yet has orbital structure permissive of electron injection at the dye-coated interface.

One of the significant problems remaining is how to optimize light absorption precisely at the dye-oxide interface in multi-component arrays. Cell efficiency is often observed to decrease when greater than monolayer coverage is attained [14,15,16,17,18], likely because the highly packed dyes, present often as poorly characterized adsorbates or stacks, are competitively quenched to the ground state before electron injection to the oxide’s conduction band can ensue. A much less extensively investigated alternative would be to explore the attachment of multiple dyes covalently bound via a well-defined, possibly dendrimeric, scaffolding that holds an explicit number of dye units at positions that permit sensitization without facilitating self-quenching.

### 1.2. Dye-Loaded Dendrimers

Dye-loaded dendrimers are one class of building blocks for such integrated photoactive structures because of their regular, but highly branched, three-dimensional architectures [19,20,21,22,23]. A variety of structural modifications are achieved for each dendritic complex by varying the core, any bridging layers, and terminal groups, resulting in a large variety of structurally graded dendritic complexes. As with integrated arrays, other potential applications for this family can be found in intriguing nanodevices for light harvesting and other bio-inspired applications [24,25].

It is possible that dye-loaded dendrimers, a class of compounds known only within the last decade or so [26], might embody the characteristics of an ideal photosensitizer. Although relatively little is known about the excited states of these materials, a study of photosensitization efficiency as a function of dendrimer size could reveal a great deal about the influence of aggregation on charge injection at solid-liquid interfaces. That is, steady-state and time-resolved emission and absorption measurements will allow us to define optimal efficiency for excited state exchange in an integrated array.

**Scheme 1:**
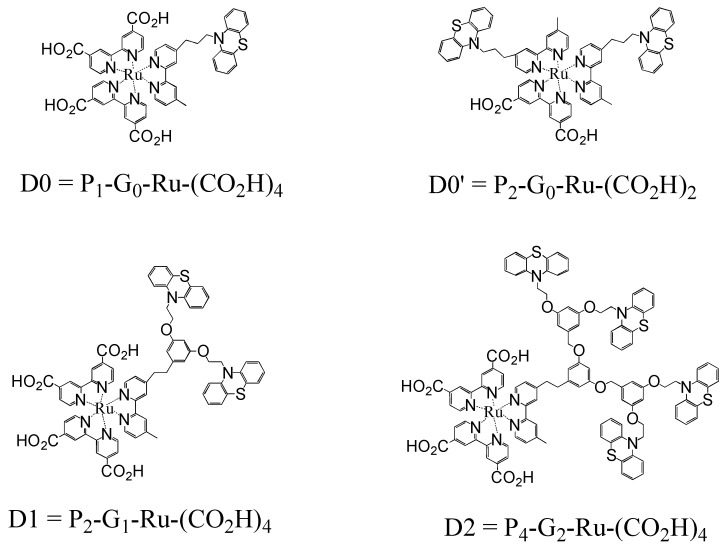
Structures of the Ru(II) dye-loaded dendrimers **D0**–**D2**.

The materials studied here were synthesized as Frechet-type dendrimers [26,27,28] modified by covalent attachment of highly absorptive dyes (phenothiazines) at each of the terminal sites onto a redox-active core (a tris-bipyridyl ruthenium (II) complex) chemically anchored to a polycrystalline TiO_2_ thin film via two or four carboxylate groups [29]. A series of photoactive dendrons having up to three generations of bridges linking the core and peripheral phenothiazines were employed, Scheme 1.

The four complexes studied, referred to as **D0**, **D0’**, **D1**, and **D2**, where the number indicates dendrimer generation, vary in structure with the general formula P_n_G_n_-Ru-(CO_2_H)_n_ where the subscripts indicate, respectively, the number of bound phenothiazines (P), the dendrimeric generation (G), and the number of carboxylic acid anchoring groups [(CO_2_H)] attached to the pyridyl ligands bound to the ruthenium core. The resulting thin layers are opaque in the ultraviolet and absorb up to about 70% of incident light in the mid-region of the visible spectrum.

### 1.3. Photosensitized Charge Injection

In this paper, we explore the possibility that newly synthesized dye-loaded dendrimers might contribute to the search for photoelectrochemical efficiency for solar-induced electron injection. The dendrimer-coated metal oxide supports employed as units in multi-component arrays might initiate a process that enhances photoresponse. If so, we can determine whether a newly synthesized family of dye-loaded dendrimers with systematically varied dye density can influence the efficiency of charge injection [29]. We are particularly interested in how conformational complexity might influence absorptivity when the multi-chromophoric dye is adsorbed onto the surface of a poised, metal oxide surface.

**Scheme 2:**
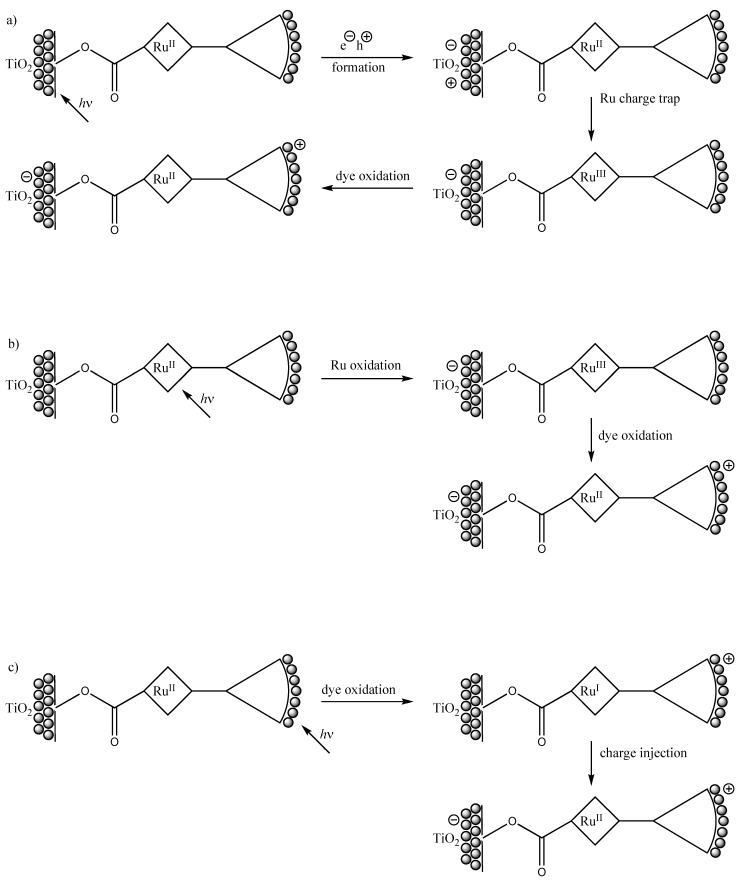
Possible photoinduced multi-step vectorial charge transfer routes in dendron/TiO_2_ composite films. Path a: band-gap photocatalysis; path b: MLCT irradiation; path c: photosensitization by surface-bound dyes.

To this end, we employ a new class of highly absorptive photoactive dendrimeric films that, when chemically bound to a TiO_2_ support, may sensitize photoinduced multistep vectorial charge injection into the conduction band of the semiconductor support, Scheme 2.

In principle, this integrated system affords three distinct possible pathways to net charge injection at the complex/TiO_2_ interface: that induced by direct band-gap excitation of the metal oxide support, Scheme 1 (path a), by metal-to-ligand charge transfer (MLCT) irradiation into the ruthenium complex (path b), or by dye photosensitization initiated by HOMO-LUMO excitation of the appended phenothiazine moieties (path c). The relative contributions of these three paths vary with incident wavelength, with the onset of path a taking place at wavelengths between about 375–400 nm, path b being observed upon photoexcitation in the visible (broad absorption around 450 nm), and path c dominating in the ultraviolet (wavelengths shorter than about 360 nm). In our work, we focus on path c, i.e., photosensitization by charge injection initiated by excitation of the highly absorptive dyes bound to the meal oxide scaffold via the dendritic linker. This focus allows us to consider the class as a biomimetic system that duplicates the multi-chromophore absorptions encountered in natural light-absorbing antennas [24-25].

Each of these sequences has been demonstrated independently in simpler non-integrated models [2,3] and references contained therein. Thus, wavelength dependence in MLCT excitation, excited state decay times, and rate constants for back electron transfer from reduced acceptors to oxidized donors or across an interfacial boundary have been characterized in analogous systems. But, to our knowledge, only a few studies of direct multi-component two-step (or more) photosensitization from multi-chromophore dye-loaded dendrimers have been reported. Nor has the spectral effect of surface-binding of such arrays on a metal-oxide semiconductor support been extensively explored. In our integrated system, photocurrent would be produced via direct excitation of appended phenothiazines, followed by excited state equilibration among dyes bound to a common dendritic linker, and ultimately by interfacial electron injection from the excited dye’s LUMO into the conduction band of the TiO_2_ photoanode.

We are specifically interested in improved efficiency of dye photosensitization attained by attaching multiple chromophores, thus achieving higher densities of dyes through higher generation number of the absorptive dendron, Scheme 1, path c. The observed rate of electron transfer between the cationic ruthenium complex and the semiconductor film surface is affected by the strength of the carboxylic acid binding as a mediator of electronic coupling, by band edge bending, and somewhat less by the dendron’s absorptivity [30,31,32]. By varying the size and absorptivity of the appended dendrons, we hoped to achieve structurally predictable gains in net long-lived charge-separation. Structural retardation of charge-recombination is also found to be increased by the larger separation between the oxidized dye and the n-doped TiO_2_ surface [20,21].

We designed these studies to address the relationship between the facility of light harvesting by the multiple dendritic dyes and the efficiency of interfacial charge transfer in trapping a photogenerated electron-hole pair by suppressing intramolecular recombination. We hoped that these measurements could provide insight into the fundamental processes that govern charge transfer dynamics at the dendritic dye sensitizer/TiO_2_ interface when mediated by a redox complex acting as an electron transfer bridge.

## 2. Results and Discussion

### 2.1. Spectral Analysis

The dendritic complexes bearing one – four phenothiazine groups were characterized by UV-visible absorption, steady state emission spectroscopy, and luminescence decay monitored at wavelengths from 570 nm to 730 nm on time scales between tens of picoseconds to many microseconds. Flash photolysis produced transient absorptions monitored at wavelengths between 370 nm to 670 nm on time scales ranging from a few nanoseconds to seconds. Laser excitations for the kinetics measurements were set at 355 nm, 400 nm, 455 nm, and 532 nm. Excitation at 355 nm avoids interference from Raman scattering, which was encountered as a minor problem with 532 nm excitation, and discriminates in favor of the appended phenothiazines and against any other species that might be present as trace impurities. Most of the data reported here are therefore derived from 355 nm excitation. In transient absorption measurements, the primary observables were bleaching at the MLCT maximum at 455 nm and a transient absorption near 530 nm [2,14,15,16,17,18]. Because it is difficult to measure transient absorption precisely at the excitation wavelength, it was essential to employ multiple excitation wavelengths in these studies.

Electrochemical measurements for the dendritic series proved to be similar to those reported previously [29], with no evidence for redox shifts in the presence of the more heavily loaded dyes, i.e., first or second generations. Although the second-generation dye-substituted dendrimer has a conformationally flexible branched dendritic structure in its higher generations, there is no evidence from absorption spectroscopy for intramolecular stacks. 

Mechanically and optically, the dye-coated films were quite robust. Films wrapped in tissue paper could be stored on the benchtop storage, but they could be scratched by determined abrasion. Optical measurements made after storing films for more than 12 months, while wrapped in aluminum foil but otherwise exposed to ambient conditions, revealed no optically detectable differences. A few thousand laser pulses of about 5 ns duration, totaling tens of joules per square centimeter irradiance, yielded no evidence for photolytic degradation. 

### 2.2. Ligands and Complexes in Homogeneous Solution

Absorption spectra of the carboxylated tris-bipyridyl ruthenium complexes substituted at the periphery with multiple phenothiazine groups, and of phenothiazine itself, were measured in deaerated methanol. UV excitation was required to produce either emission or transient absorptions. Emission measurements indicate excited state lifetimes near 1 ns, and transient absorption was observable throughout the visible region, consistent with the temporal behavior expected for a sensitized MLCT triplet state.

The probe compounds in methanol showed prominent MLCT absorptions in the blue region, along with dye-localized transitions in the ultraviolet. Luminescence decayed as single exponentials with emission lifetimes near 1 μs in the absence of oxygen and near 200 ns when aerated. Flash photolysis was followed by bleaching of the MLCT blue absorption along with a transient absorption at both shorter and longer wavelengths, all with the same lifetime as the emission. 

**Figure 1:**
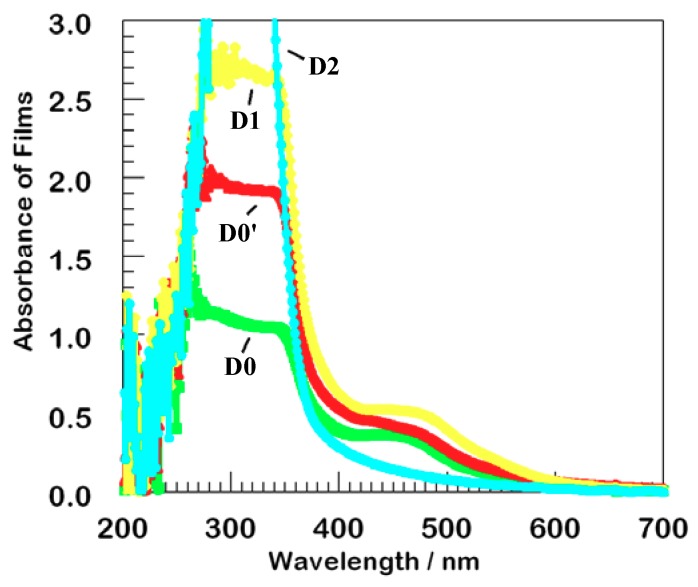
Absorption of thin films of the series of dendrimers on TiO_2_. The second generation film, **D2,** has the strongest absorption at 300 nm but the least absorption at 450 nm. Color coding remains the same in all figures.

The excited state characteristics of the **D1** and **D2** dendrimers were similar to the parent **D0** with two exceptions: (1) the ratio of absorption in the UV to that in the MLCT region increased in the presence of multiple phenothiazine moieties, Figure 1; and (2) all dendritic complexes exhibited strongly non-exponential decays for emission, transient absorption, and bleaching, Figure 4 [26].

### 2.3. Steady State Optical Properties of Films 

Figure 1 shows absorption spectra (without normalization) for four typical films of the dendrimers bound to an optically transparent TiO_2_ film, collected with the excitation beam perpendicular to the surface. The metal-to-ligand charge transfer (MLCT) region exhibits similar absorption to that observed in solution, about 0.45 AU near 450 nm. A local density of about 200 ruthenium atoms per square nanometer on the titanium dioxide surface translates to single monolayer coverage, assuming that the same extinction coefficient is observed in the film as in methanolic solution.

Upon excitation at 355 nm, nearly all of the incident light was absorbed by the dyes, which have a ten-fold higher absorption than TiO_2_ at that wavelength, so minimal incident light can be transmitted through the attached absorptive layer to the TiO_2_ substrate. That is, there is only negligible direct electron-hole separation via path a, Scheme 1. All of these films were therefore suitable for thin film sensitization studies. We hypothesis that the stronger absorption by **D2** at 300 nm but less at 450 nm likely reflects three-dimensional structural flexibility.

Absorption in the mid-UV is dominated by the appended dye chromophores. Absorption increases with dendrimer generation, as expected. In fact, the **D2** film has the greatest absorption because of its larger number of appended phenothiazines. The observed hypsochromic shift in the series clearly results from dye aggregation consequent to multiple conformations in the dye-loaded dendrimeric dyes.

**Figure 2:**
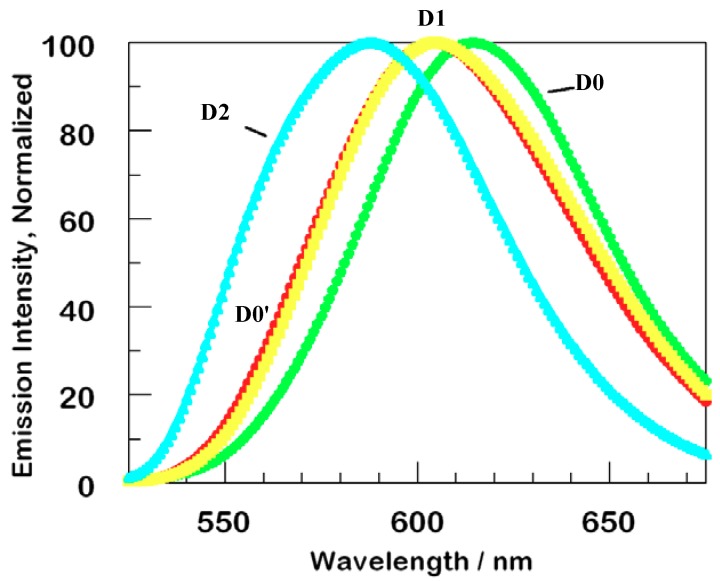
Normalized steady state luminescence of thin films of **D0-D2** series dendrimers. The second generation dendrimer, **D2,** has its maximum at the shortest wavelength and the parent **D0** has its at the longest wavelength. Excitation wavelength was 355 nm, normalization was accomplished by comparing relative absorption calculated using Beer’s Law at 355 nm.

Steady state luminescence measurements upon excitation at 355 nm are displayed in Figure 2 [2,12]. The films were positioned at about 45° from the incident beam, and glass filters were used to eliminate specular and diffuse reflection in the spectrofluorimeter. At this wavelength, direct absorption and excitation is localized in the phenothiazine dyes. The emission spectra in Figure 2 are normalized, in order to emphasize the most striking feature, a pronounced hypsochromic shift between **D0** and **D2**. Although emission profiles from **D1** and **D0′** are quite similar, they are shifted substantially from the **D0** parent, and **D2** is shifted as much again. 

We assign these shifts to steric distortion due to a combination of chromophore density in the dendrimer itself and to minor complex layering. Before normalization, the intensity of luminescence for the films **D0**, **D0’**, **D1**, **D2** is in the ratio of about 1, 3, 4, 6, i.e., roughly proportional to the number of appended dyes per complex. 

The slightly lower absorptivity of **D0** accounts for only a minor portion of the small luminescence of that sample; there appears to be greater bimolecular trapping and self-quenching in this, the least highly loaded film. The high emission yield for **D2** is even more notable: external and aggregative quenching must be reduced in this, the most heavily loaded of the films.

### 2.4. Time-Resolved Photoluminescence of the Thin Films

**Figure 3:**
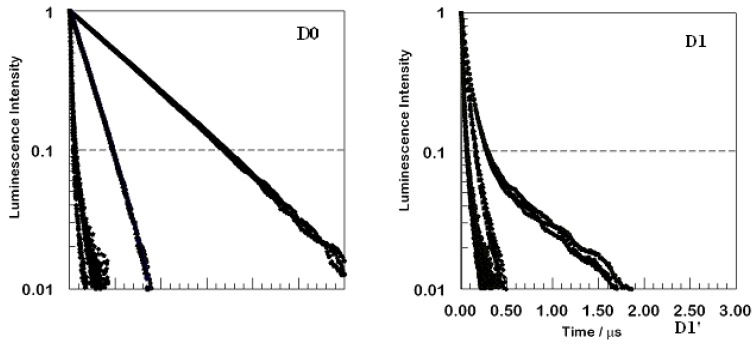
Comparison for two compounds, **D0** and **D1**, of time-resolved luminescence from argon-sparged methanolic solution (top set of curves in each panel), from the same solutions equilibrated with air (middle set of curves), and from thin films (lowest and fastest decaying set of curves). Excitation wavelength was 355 nm.

All films showed strong quenching of luminescence. This is evident from the weak steady-state luminescence of the films compared with the solutions from which the surface-bound complexes were equilibrated. Time-resolved luminescence decay measurements capture any extremely fast decay (tens of picoseconds or less) from static quenching. This is illustrated in Figure 3, which compares luminescence decay in solution to decay in films for **D0**, with a single phenothiazine electron donor, and for **D1**, the first generation dendrimer.

The top panel shows that the decay of **D0** is rapid in oxygenated solution, but is reduced significantly in the absence of air. The normalized decay kinetics are first order in solution, with or without oxygen present, but not in the film. The first generation dendrimer **D1** in the lower panel shows non-exponential luminescence decay, even in solution. The observed signal amplitude decays much more rapidly than was the case for **D0** in solution. Decay remains non-exponential and becomes faster yet for aerated solutions and, especially, for the film. Each panel includes three emission wavelengths overlaid. The kinetic traces displayed are for wavelengths of 610 nm, 630 nm, and 660 nm, which span the broad emission peak. The decays are nearly indistinguishable, showing slight, if any, variation for different wavelengths in solution. For the films, there appears to be some slight variation, with faster decays at the shorter wavelengths. The same behavior, i.e., strong quenching in the films relative to that observed in methanol solution and a highly non-exponential behavior in all films along with wavelength variation only in the films, held for all four species investigated **D0−D2**. 

The curves in Figure 3 were all obtained using a nanosecond pulse laser, so the decays for the films are instrumentally limited; in reality, one component of the observed decay is even faster than it appears in Figure 1. An adequate analysis of the decays of the films always required at least three exponentials to achieve a good fit. Very likely, a continuous distribution of lifetimes is observed. 

**Figure 4:**
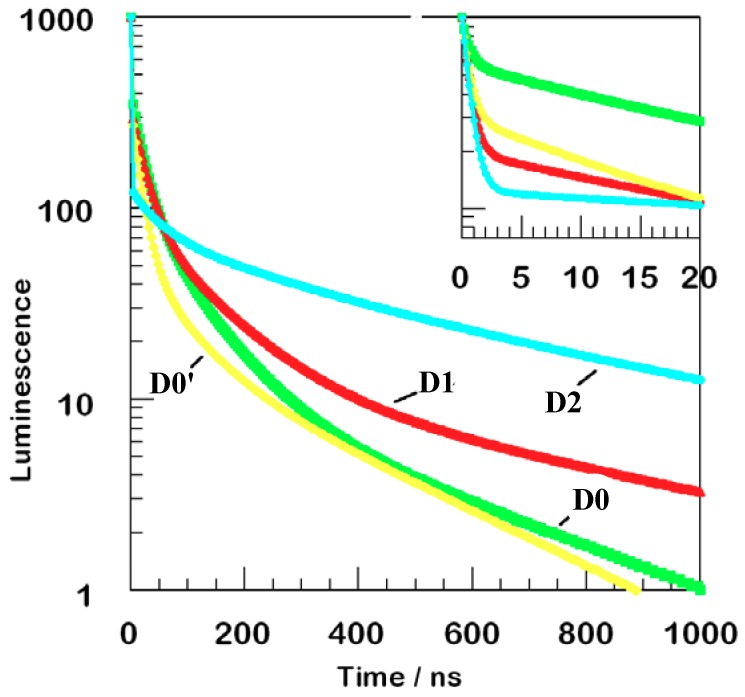
Luminescence decay kinetics near 650 nm or 660 nm for four films. The main curve shows data normalized upon 355 nm excitation. The inset shows more detail of faster decay measured upon femtosecond laser excitation at 400 nm. The vertical scales are identical: only the horizontal range is different for the inset.

Figure 4 examines more closely the kinetics of luminescence decay ensuing from excitation at 355 nm using a semilog plot for the films of all four members of the dendrimer series that appear in Scheme 2.

Only one wavelength is monitored for each film, either 650 nm or 660 nm. These data are composites of measurements acquired using picosecond and nanosecond data. The initial portion of the decay, so fast as to be non-exponential, is fit therefore with a distribution of sub-nanosecond time constants. The picosecond data displayed in the inset are a smoothed fit to a single exponential with a 600 ps time constant. At least 50% to 90% of the initial amplitude decays on a sub-nanosecond time scale. If a substantial fraction of the decay is much faster than 600 ps, an even larger fraction of the initial excitation disappears from the emitting state. 

Another decay pathway is even slower, 20 ns to 50 ns typically, probably reflecting direct electron transfer to substrate, but with less effective coupling, perhaps because of slow transport among the dyes bound within the monolayer film. The remarkable difference in the long-lived components of the observed decay is likely the result of the 355 nm incident photons localized in the appended dyes, Scheme 1, path c. 

In every case, luminescence is profoundly non-exponential, Figure 4. A major fraction is extremely fast, and there are measurable contributions from at least three more components. Although the fraction of fast decay varies from film to film, a broader range is observed for the more highly substituted dendrimers, demonstrating that dye-loading does indeed affect the observed kinetics.

**Figure 5:**
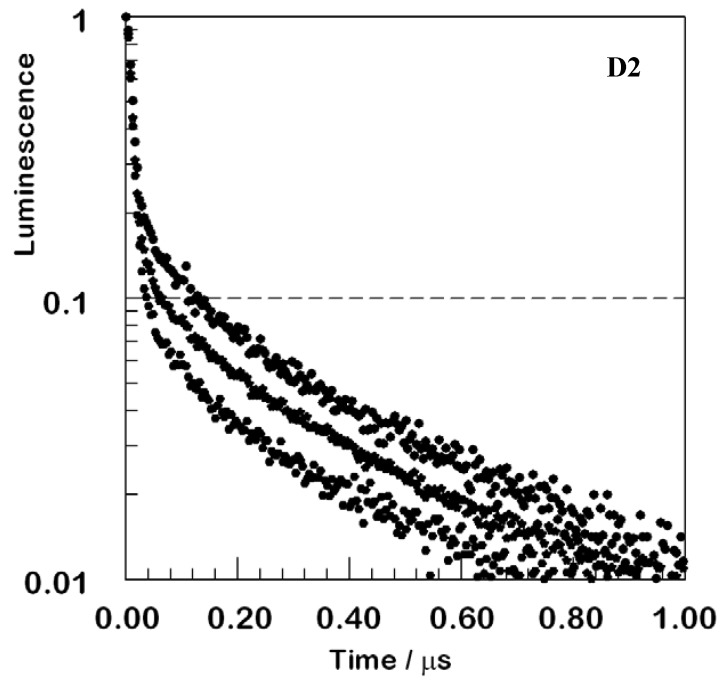
Normalized luminescence decays for the **D2** film at three emission wavelengths. Top, 610 nm; middle, 630 nm; bottom, 660 nm; all upon excitation at 355 nm. The trend continues to shorter and longer wavelengths, but signal-to-noise deteriorates in the wings so that overlaid display would be ineffective.

In Figure 5, **D2** luminescence is probed to illustrate the wavelength-dependent variation of luminescence decay in the film. At shorter wavelengths, a larger fraction of the emission remains unquenched and persists for long times.

### 2.5. Transient Absorption

**Figure 6:**
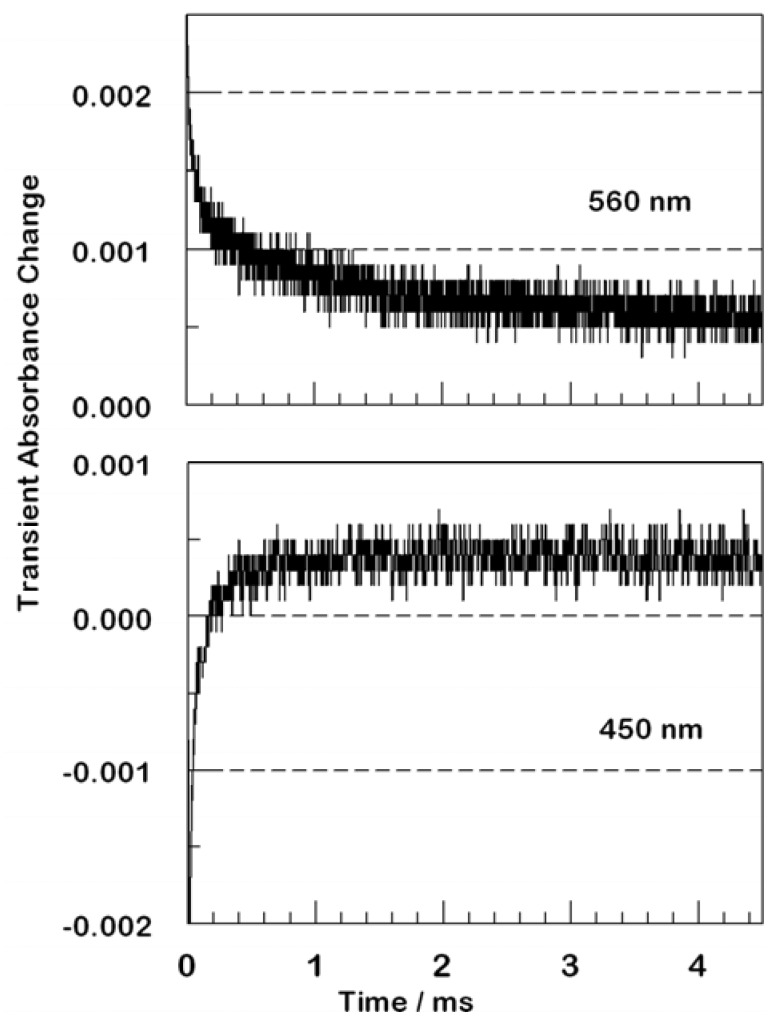
Transient kinetic absorption traces following nanosecond excitation of a **D2** film. The upper panel had 355 nm excitation with 560 nm detection. The transient is absorptive at all times and does not return to baseline on this timescale. The lower panel had 355 nm excitation with 450 nm detection. At early times, bleaching characteristic of excited or oxidized ruthenium is observed, but this converts to absorption after tens to hundreds of microseconds. The absorption persists to much longer times.

Efficient electron transfer quenching deriving from electron transfer from the excited dyes to the conduction band of the metal oxide support is less efficient for the larger dendrimers. The dendrimers decrease the rate of back electron transfer and increase the duration of the charge-separated state. Upon flash photolysis of a **D2** film, transient absorptions are consistently produced, Figure 6, with lifetimes near or slightly longer than 1 ms. This is true for excitations throughout the visible region from 370 nm to 670 nm, probably arising from oxidized phenothiazine moieties. Decay curves measured at longer times than shown in Figure 6 reveal that this absorption feature decays to baseline with complex kinetics. At least two exponentials are required for an acceptable fit, one at about 20 ms and a second at about 300 ms.

The **D1** film behaves similarly, as does **D0′**, and the largest dendrimer **D2** exhibits even slower recombination kinetics. The parent with a single phenothiazine **D0** has a negligible fraction of persistent charge-separation. At shorter times, all films show bleaching in the MLCT ground state absorption [25,26], a feature seen also with the coordination compounds in solution. 

Changes in the time scale of luminescence are attributed to electron transfer from the excited dye via the Ru(II) complex bound through the carboxylates to the TiO_2_ substrate, followed by additional changes attributed to suppression of back electron transfer to the oxidized phenothiazine from the reduced semiconductor surface [20]. Decay taking place over a range of a few hundred microseconds (seen in Figure 6) are likely associated with such intramolecular processes, or they may reflect, in part, some elements of faster charge recombination via electron transfer from the conduction band of TiO_2_ back to oxidized phenothiazine [20].

At wavelengths removed from the peak MLCT ground-state absorption, the transient spectra are absorptive at all times. Isosbestic points indicate interconversion of the species responsible for transient absorption and transient bleaching at nanosecond times, upon excitation between 410 nm and 490 nm. The observation that all kinetic decays are highly non-exponential establishes that multiple pathways exist in this series for charge injection into the metal oxide semiconductor support.

## 3. Experimental 

### 3.1. Chemicals

All chemicals were purchased from Aldrich. Potassium carbonate (K_2_CO_3_) was dried in an oven at 130 °C. Methylene chloride (CH_2_Cl_2_) and tetrahydrofuran (THF) were distilled from CaH_2_ and sodium/benzophenone, respectively. All other reagents were used as received. NMR spectra were recorded in CDCl_3_ unless otherwise noted and are reported as chemical shifts in ppm *vs* TMS. Bis[2,2’-bipyridine-4,4’-carboxylic acid (dcb)] ruthenium(II) was prepared according to a literature procedure [34].

### 3.2. Preparation of Dye-loaded Dendrons Bound to Bis[2,2’-bipyridine-4,4’-dicarboxylic acid (dcb)]ruthenium Complex [P_n_G_n_-Ru-(CO_2_H)_4_]

The intended dendron [P**_n_G_n_**-Bipy] (1.0 equiv) [34] and Ru(dcb)2∙Cl2 (1.0 equiv) were heated to 120 °C–130 °C in a small amount of degassed ethylene glycol for 2 days. The solution was cooled and poured into about a 10-fold excess of water, and the red precipitate was collected by filtration. The crude product was loaded onto a silica gel column and eluted with 20:1 CH_2_Cl_2_:methanol. A bright orange solution with red luminescence was collected and the solvent was removed under vacuum to afford the ruthenated dendron. The complex D0′ P_2_G_0_Ru(CO_2_H)_2_ was prepared by the route previously described [30].

**D0 = P_1_G_0_Ru(CO_2_H)_4_:** yield 60% , Anal. Calcd for C_50_H_39_Cl_2_N_7_O_8_RuS∙xH_2_O: C, 56.13; H, 3.67; N, 9.16, found: C, 55.84, H, 3.92; N, 8.84. FAB MS, m/z = 999.0 [M – 2Cl].
**D1 = P_2_G_1_Ru(CO_2_H)_4_:** yield 30% , Anal. Calcd. for C_71_H_56_Cl_2_N_8_O_10_RuS_2_∙xH_2_O: C, 60.17; H, 3.98; N, 7.91, found: C, 59.62, H, 4.07; N, 7.64. FAB MS, m/z = 1368.2 [M + Na – H – 2Cl].
**D2 = P_4_G_2_Ru(CO_2_H)_4_:** yield 10%, Anal. Calcd. for C_113_H_90_Cl_2_N_10_O_14_RuS_4_∙xH_2_O: C, 64.26; H, 4.29; N, 6.63, found: C, 61.54; H, 4.35; N, 6.64. MALDI-TOF-MS (matrix: DHB), m/z = 2061.5 [M + Na–2H–2Cl].

### 3.3. Preparation of Dendron/ TiO_2_ Nanocomposite Films 

The porous TiO_2_ nanocrystalline films deposited on glass were prepared by the method of Zhang *et al* [36]. The average particle diameter was approximately 9 µm, and the average thickness was ~ 10 µm. The attachment of the **P_n_G_n_Ru(CO_2_H)_n_** dendrons to the nanocrystalline TiO_2_ films was achieved by immersion of the glass film in an ethanolic solution of the dendron at room temperature overnight. Each of the dye-modified films **D0 – D2** showed strong adsorption to the surface of the TiO_2_ film as was determined by the lack of bleaching of the deeply colored dye overnight. 

### 3.4. Steady-state Absorption and Luminescence 

Absorption spectra were measured using an Agilent 8453 spectrophotometer. Steady state luminescence was measured using a Jasco FP-6200 spectrofluorimeter.

### 3.5. Time-Resolved Emission

Lifetimes were measured on a nanosecond laser apparatus with a frequency-multiplied Nd:YAG or XeCl excimer-pumped dye to produce excitation at 355 nm, 455 nm, or 532 nm. The monochromator used a 10 nm bandpass; glass filters were added to eliminate scattered excitation. Digitization was accomplished on a LeCroy 9361 digital oscilloscope. Nonlinear least squares curve fitting was accomplished with software written in-house. Lifetimes were measured with a femtosecond Ti-sapphire laser for excitation and coupled with instrumentation for time-correlated single photon counting, deconvoluted to obtain time resolutions below 1 ns [35].

### 3.6. Transient Absorption

Absorption changes produced by nanosecond flash photolysis were measured on the same nanosecond apparatus, except that a probe beam traversed the sample and was detected by the photomultiplier. Nanosecond laser pulse fluxes were in the range 10−100 mJ cm^−2^, about an order of magnitude more concentrated than for emission studies. The excitation and probe beams were co-linear. A continuous tungsten halogen lamp and a pulsed xenon discharge (500 μs full width at half max) were used for small signals at high precision over a range of about 10 μs. For any given time range, care was taken to optimize pre-digitization electronic filtering to maximize signal-to-noise without distorting the signal.

## 4. Conclusions 

This research establishes the viability of dendrimeric multichromophoric arrays as efficient as efficient biomimetic sensitizers. We conclude that dye-loaded dendrimers can indeed be efficient photosensitizers in integrated arrays. Thus, each of the proposed routes shown in Scheme 2 can be independently observed if proper excitation wavelengths are used. Conformational complexity of heavily-loaded sensitizers opens up competing electron injection routes and, hence, optical and kinetic complexity. Nonetheless, Dye-loaded dendrimers of differing dendrimer generation influence the efficiency of charge injection [30]. Indeed, conformational complexity affects as well the observed absorptivity and chromophore lifetime when the dendritic multi-chromophoric dye functions as a photosensitizer in a photoelectrochemical cell. As a result, these synthetic aggregates are excellent components for complex photochemical arrays.

## References

[1] Durr H., Bossman S. (2001). Ruthenium polypyridine complexes: On the route to biomimetic assemblies as models for the photosynthetic reaction center. Acc. Chem. Res..

[2] Chanon M., Fox M.A. (1986). Photoinduced Electron Transfer.

[3] Vlcek A., Balzani V. (2001). Electron Transfer in Chemistry.

[4] Meyer T.J. (1989). Chemical approaches to artificial photosynthesis. Acc. Chem. Res..

[5] Grätzel M. (1983). Energy Resources through Photochemistry and Catalysis.

[6] Wenger B., Grätzel M., Moser J.E. (2005). Rationale for kinetic heterogeneity of ultrafast light-induced electron transfer from Ru(II) complex sensitizers to nanocrystalline TiO_2_. J. Am. Chem. Soc..

[7] O’Reagan B., Grätzel M. (1991). A low-cost, high-efficiency solar cell based on dye-sensitized colloidal TiO_2_ films. Nature.

[8] Gust D., Moore T.A., Moore A. (2001). Mimicking photosynthetic solar energy transduction. Acc. Chem. Res..

[9] Bard A.J., Fox M.A. (1995). Artificial photosynthesis: Solar splitting of water to hydrogen and oxygen. Accts. Chem. Res..

[10] Fujishima A., Honda K. (1971). Electrochemical evidence for the mechanism of the primary stage of photosynthesis. Bull. Chem. Soc. Japan.

[11] Fujishima A., Honda K. (1972). Electrochemical photolysis of water at a semiconductor electrode. Nature.

[12] Rajeshwar K. (2007). Hydrogen generation at irradiated oxide semiconductor–solution interfaces. J. Appl. Electrochem..

[13] Nozik A.J. (1977). Photochemical diodes. Appl. Phys. Lett..

[14] Gao F., Wang Y., Shi D., Zhang J., Wang M.K., Jing X.Y., Humphry-Baker R., Wang P., Zakeeruddin S.M., Grätzel M. (2008). Enhance the optical absorptivity of nanocrystalline tio2 film with high molar extinction coefficient ruthenium sensitizers for high performance dye-sensitized solar cells. J. Am. Chem. Soc..

[15] Moore J.S. (2008). Molecular design of thin film optoelectronic materials for solar cells. J. Am. Chem. Soc..

[16] O’Reagan B.C., Lopez-Duarte I., Victoria-Martinez M., Fomell A., Alero J., Morandeza A., Palomares E., Terres T., Durant J.R. (2008). Catalysis of recombination and its limitation on open circuit voltage for dye sensitized photovoltaic cells using phthalocyanine Dyes. J. Am. Chem. Soc..

[17] Dance Z.E.X, Ahrens M.J., Vega A.M., Ricks A.B., McCamani D.W., Ratner M.A., Wasielewski M. (2008). Direct observation of the preference of hole transfer over electron transfer for radical ion pair recombination in donor-bridge-acceptor molecules. J. Am. Chem. Soc..

[18] Veldman D., Ipek O., Maskers S.E.J., Sweetssen J., Koelse M.M., Veenstra S.C., Kroon J.M., vanVeldman S., Loos J., Janssen R.A. (2008). Compositional and electric field dependence of the dissociation of charge transfer excitons in alternating polyfluorene copolymer/fullerene blends. J. Am. Chem. Soc..

[19] Stewart G.M., Fox M.A. (1996). Chromophore-Labeled Dendrons as light harvesting antennae. J. Am. Chem. Soc..

[20] Ghaddar T.H., Whitesell J.K., Fox M.A. (2001). Excimer formation in a naphthalene-labeled dendrimer. J. Phys. Chem..

[21] Ghaddar T.H., Wishart J.F., Thompson D.W., Whitesell J.K., Fox M.A. (2002). A dendrimer-based electron antenna: paired electron-transfer reactions in dendrimers with a 4,4’-Bipyridine core and naphthalene peripheral groups. J. Am. Chem. Soc..

[22] Zhu L.Y., Tong X.F., Li M.Z., Wang E.J. (2001). Luminescence enhancement of Tb^3+^ ion in assemblies of amphiphilic linear-dendritic block copolymers: Antenna and microenvironment effects. J. Phys. Chem. B.

[23] Taranekar P., Fulghum T., Patton D., Ponnapati R., Clyde G., Advincula R. (2007). Investigating carbazole jacketed precursor dendrimers: Sonochemical synthesis, characterization, and electrochemical crosslinking properties. J. Am. Chem. Soc..

[24] Jiang D.L., Aida T. (1997). Photoisomerization in dendrimers by harvesting of low-energy photons. Nature.

[25] Devadoss C., Bharathi P., Moore J.S. (1996). Energy Transfer in dendritic macromolecules: Molecular size effects and the role of an energy gradient. J. Am. Chem. Soc..

[26] Sharma V. (2009). Structural origin of circularly polarized iridescence in jeweled beetles. Science.

[27] Frechet J.M.J. (1994). Functional polymers and dendrimers: Reactivity, molecular architecture, and interfacial energy. Science.

[28] Hawker C.J., Frechet J.M.J. (1990). Preparation of polymers with controlled molecular architecture: A new convergent approach to dendritic macromolecules. J. Am. Chem. Soc..

[29] Wooley K.L., Hawker C.J., Frechet J.M.J. (1993). Unsymmetrical three-dimensional macromolecules: preparation and characterization of strongly dipolar dendritic macromolecules. J. Am. Chem. Soc..

[30] Zhu L., Magde D., Whitesell J.K., Fox M.A. (2009). Optical and electrochemical properties of shell-core dendrimers: Ruthenium coordination complexes capped with sized phenothiazine-substituted bipyridines. Inorg. Chem..

[31] Biju V., Micic M., Hu D.H., Lu H.P. (2004). Intermittent single-molecule interfacial electron transfer dynamics. J. Am. Chem. Soc..

[32] Haque S.A., Handa S., Peter K., Palomares E., Thelakkat M., Durrant J.R. (2005). Supermolecular control of charge transfer in dye-sensitized nanocrystalline TiO_2_ films: Towards a quantitative structure-function relationship. Angew. Chem. Int. Ed..

[33] Argazzi R.A., Bignozzi C.A., Heimer T.A., Castellano F.N., Meyer G.J. (1997). Light-induced charge separation across Ru(II)-modified nanocrystalline TiO_2_ interfaces with phenothiazine donors. J. Phys. Chem. B.

[34] Odobel F., Zabri H. (2005). Preparations and characterizations of bichromophoric systems composed of a ruthenium polypyridine complex connected to a difluoroborazaindacene or a Zinc phthalocyanine chromophore. Inorg. Chem..

[35] Nazeeruddin M., Zakeeruddin S.M., Humphry-Baker R., Jirousek M., Liska P., Vlachopoulos N., Shklover V., Fischer C.H., Grätzel M. (1999). Acid-base equilibria of (2,2’-Bipyridyl-4,4’-dicarboxylic acid) ruthenium(II) complexes and the effect of protonation on charge-transfer sensitization of nanocrystalline titania. Inorg. Chem..

[36] Zhang D., Downing J.A., Knorr F.J., McHale J.L. (2006). Room-temperature preparation of nanocrystalline TiO_2_ films and the influence of surface properties on dye-sensitized solar energy conversion. J. Phys. Chem. B.

